# SARS-CoV-2 Prevalence among Outpatients during Community Transmission, Zambia, July 2020

**DOI:** 10.3201/eid2708.210502

**Published:** 2021-08

**Authors:** Jonas Z. Hines, Sombo Fwoloshi, Davies Kampamba, Danielle T. Barradas, Dabwitso Banda, James E. Zulu, Adam Wolkon, Samuel Yingst, Mary Adetinuke Boyd, Mpanji Siwingwa, Lameck Chirwa, Muzala Kapina, Nyambe Sinyange, Victor Mukonka, Kennedy Malama, Lloyd B. Mulenga, Simon Agolory

**Affiliations:** US Centers for Disease Control and Prevention, Lusaka, Zambia (J.Z. Hines, D.T. Barradas, A. Wolkon, S. Yingst, M.A. Boyd, S. Agolory);; University Teaching Hospital, Lusaka (S. Fwoloshi, D. Kampamba, M. Siwingwa, L. Chirwa, L.B. Mulenga);; Zambia Ministry of Health, Lusaka (S. Fwoloshi, K. Malama, L.B. Mulenga);; Zambia Field Epidemiology Training Program, Lusaka (D. Banda, J.E. Zulu);; Zambia National Public Health Institute, Lusaka (M. Kapina, N. Sinyange, V. Mukonka)

**Keywords:** coronavirus disease, disease control strategies, disease transmission, pandemics, real-time PCR, respiratory infections, SARS-CoV-2, severe acute respiratory syndrome coronavirus 2, whole genome sequencing, Zambia, zoonoses

## Abstract

During the July 2020 first wave of severe acute respiratory syndrome coronavirus 2 in Zambia, PCR-measured prevalence was 13.4% among outpatients at health facilities, an absolute difference of 5.7% compared with prevalence among community members. This finding suggests that facility testing might be an effective strategy during high community transmission.

The first cases of infection with severe acute respiratory syndrome coronavirus 2 (SARS-CoV-2), the virus that causes coronavirus disease (COVID-19), were reported in Zambia in March 2020 (*1*). During the first wave of infections, confirmed cases rapidly increased during July and peaked in August 2020 (Appendix). Zambia initially focused on screening travelers at points-of-entry and tracing contacts of persons with laboratory-confirmed cases. In April 2020, the Zambia Ministry of Health began SARS-CoV-2 surveillance among hospital inpatients and outpatients to identify cases of local transmission (*1*,*2*). It was believed that testing in health facilities would be more efficient at identifying cases than testing in the general population, which was particularly noteworthy given the severe shortage of SARS-CoV-2 tests in Africa early in the pandemic (*3*,*4*). A household prevalence survey conducted in 6 districts in Zambia in July 2020 found a community SARS-CoV-2 prevalence of 7.6% by using real-time PCR (rPCR) (*5*). To determine if facility testing was an effective case-finding strategy during a period of high community transmission, we compared SARS-CoV-2 prevalence among outpatients, overall and stratified by reasons for visiting the facility, with prevalence among community members in the same districts (*5*).

## The Study

During July 2–31, 2020, we administered a cross-sectional prevalence survey of patients attending 20 outpatient clinics, including hospitals and health centers, in 6 districts in Zambia (Appendix). The number of facilities we selected in each district was proportional to the number of facilities in the other districts (Appendix). We recruited participants from outpatient departments regardless of their reason for visiting the facilities. Study teams were instructed to recruit ≥50 participants per facility and to attempt to show no preference in selection. We obtained consent or assent (for minors) before beginning study procedures. Participants completed an interviewer-administered questionnaire that included demographics, medical history, SARS-CoV-2 exposures, history of recent illness, and reason for visiting the facility. Concurrently, we conducted a cluster-sampled household prevalence survey in the same 6 districts (*5*). These surveys provided an opportunity to directly compare outpatient and community SARS-CoV-2 prevalence estimates. The study was approved by the Zambia National Health Research Authority and the University of Zambia Biomedical Research Ethics Committee. The activity was reviewed by the US Centers for Disease Control and Prevention (CDC) and conducted consistent with applicable US federal law and CDC policy.

We tested nasopharyngeal specimens for SARS-CoV-2 RNA by using rPCR and plasma specimens for SARS-CoV-2 antibodies by using ELISA. We extracted RNA for rPCR using the QIAGEN Viral Mini procedure (https://www.qiagen.com). We used the Maccura SARS-CoV-2 Fluorescent PCR kit (https://www.maccura.com) on the QuantStudio 3 platform (ThermoFisher, https://www.thermofisher.com) as the primary rPCR diagnostic (*6*) and used the CDC assay method to confirm positive and indeterminant results (*7*). We performed the Euroimmun ELISA (PerkinElmer, https://www.perkinelmer.com) to test for spike protein IgG in single replicate (*8*). Participants could take part in any or all of the survey, rPCR testing, or serologic testing options based on personal preference.

We calculated SARS-CoV-2 prevalence as the number of positive results divided by the total number of tests conducted. Estimates were calculated separately for rPCR and ELISA results. We adjusted variance and 95% CIs for clustering by facility and seroprevalence for imperfect assay test characteristics (sensitivity 64.2%; specificity 100%; L. Steinhardt, pers. comm., email, 2021 Apr 2) using the Rogan-Gladen method (Appendix). To assess various factors associated with SARS-CoV-2 prevalence among outpatients, we used bivariate Poisson regression models to calculate prevalence ratios (PRs) and 95% CIs, with a random effects term for facility.

Of 1,975 persons representing ≈3.5% of ≈57,000 outpatients from the 20 facilities that we approached in July 2020 about participating (District Health Information System version 2; https://dhis2.org), 1,952 (98.8%) completed the questionnaire and 1,908 (97.7%) submitted either nasopharyngeal (1,490; 76.3%) or blood (1,657; 84.3%) specimens or both (Appendix). Of the 1,952 total participants, the number per district ranged from 160 (8.2%) in Nakonde District to 639 (32.8%) in Lusaka District; the median number of participants per facility was 93 (interquartile range 78–107; Table 1). Median participant age was 32 years (interquartile range 24–43 years); 60.5% were female. Overall, 34.1% of participants reported having a coexisting medical condition. Fever or respiratory complaints accounted for 28.2% of reasons for visiting the facility; 2.3% of participants were specifically seeking COVID-19 testing.

**Table 1:** Severe acute respiratory syndrome coronavirus 2 prevalence measured by PCR and ELISA and prevalence ratios in study of prevalence among outpatients during community transmission, Zambia, July 2020

Characteristic	Overall proportion, n = 1,952				rPCR-measured prevalence, n = 1,490				ELISA-measured prevalence,† n = 1,657	
	No. (%)		95% CI		% (95% CI)		PR		% (95% CI)	PR
Overall	1,952		NA		13.4 (8.3–18.5)		Not applicable		8.2 (5.1–11.4)	Not applicable
Sex										
M	767 (39.4)		35.4–43.5		14.5 (8.9–20.0)		Referent		8.1 (5.0–11.2)	Referent
F	1,178 (60.6)		56.5–64.6		12.8 (7.1–18.5)		0.9 (0.7–1.1)		6.8 (3.6–10.0)	0.8 (0.6–1.2)
Age range, y										
0–9	41 (2.1)		0.9–3.4		6.3 (0.0–16.0)		Referent		13.0 (0.0–28.5)	Referent
10–19	161 (8.3)		6.0–10.6		8.2 (1.7–14.7)		1.3 (0.3–5.0)		11.0 (1.2–20.9)	0.9 (0.3–2.7)
20–29	628 (32.3)		28.5–36.2		14.0 (7.1–20.9)		2.2 (0.7–7.3)		5.3 (2.0–8.6)	0.4 (0.2–1.0)
30–39	488 (25.1)		22.7–27.6		15.4 (9.9–20.9)		2.5 (0.7–8.5)		5.7 (2.3–9.0)	0.4 (0.2–1.3)
40–49	293 (15.1)		12.0–18.1		13.2 (7.7–18.8)		2.1 (0.6–7.2)		8.4 (4.0–12.8)	0.7 (0.2–2.2)
50–59	185 (9.5)		7.1–12.0		12.2 (5.0–19.4)		2.0 (0.5–7.8)		10.2 (2.4–17.9)	0.8 (0.3–2.2)
60–69	83 (4.3)		2.9–5.7		14.7 (5.6–23.8)		2.4 (0.6–9.4)		6.5 (0.0–13.7)	0.5 (0.1–2.3)
≥70	64 (3.3)		2.0–4.6		11.1 (3.7–18.5)		1.8 (0.4–7.1)		15.6 (2.0–29.1)	1.2 (0.3–5.0)
District										
Kabwe	249 (12.8)		0.0–28.2		3.8 (1.6–6.1)		0.2 (0.1–0.4)		3.0 (2.0–4.1)	0.3 (0.2–0.5)
Livingstone	307 (15.8)		0.0–35.8		23.4 (15.5–31.4)		1.3 (0.8–1.9)		3.9 (1.4–6.4)	0.4 (0.2–0.8)
Lusaka	639 (32.8)		8.6–57.1		18.8 (13.1–24.4)		Referent		9.9 (5.5–14.4)	Referent
Nakonde	160 (8.2)		0.0–20.4		7.0 (4.5–9.4)		0.4 (0.2–0.6)		16.4 (2.5–30.3)	1.6 (0.7–4.0)
Ndola	288 (14.8)		0.0–31.9		14.1 (0.0–28.7)		0.8 (0.3–2.0)		4.8 (2.3–7.4)	0.5 (0.3–0.9)
Solwezi	304 (15.6)		0.0–33.6		6.0 (2.2–9.8)		0.3 (0.2–0.6)		4.3 (0.6–8.0)	0.4 (0.2–1.0)
Education										
Primary	476 (24.7)		19.7–29.6		10.5 (5.6–15.4)		Referent		5.2 (1.3–9.1)	Referent
Secondary	918 (47.6)		39.6–55.5		12.7 (7.7–17.7)		1.2 (1.0–1.5)		8.0 (3.6–12.5)	1.5 (0.7–3.4)
Higher	443 (23.0)		13.6–32.3		18.4 (9.6–27.1)		1.8 (1.1–2.9)		8.1 (3.6–12.6)	1.6 (0.7–3.3)
None	90 (4.7)		2.9–6.5		11.1 (1.6–20.6)		1.1 (0.6–1.8)		7.2 (0.0–14.8)	1.4 (0.6–3.5)
Don't know	3 (0.2)		0.0–0.4		0.0		Not calculated		0.0 (0.0–0.0)	Not calculated
Health facility type										
Hospital	1,127 (57.9)		33.7–82.1		16.3 (9.5–23.0)		1.7 (0.8–3.4)		7.8 (4.4–11.1)	1.2 (0.6–2.6)
Tertiary hospital	260 (13.4)		0.0–29.6		8.2 (1.7–14.7)		Not calculated		9.0 (1.6–16.3)	Not calculated
Referral hospital	381 (19.6)		0.0–41.8		16.3 (2.0–30.6)		Not calculated		4.6 (4.4–4.8)	Not calculated
Community hospital	486 (25.0)		1.8–48.1		21.3 (16.3–26.4)		Not calculated		9.2 (4.2–14.2)	Not calculated
Health center	820 (42.1)		17.9–66.4		9.7 (3.3–16.0)		Referent		6.4 (1.8–11.1)	Referent
Urban health center	738 (37.9)		14.4–61.4		9.8 (2.7–17.0)		Not calculated		6.1 (0.9–11.4)	Not calculated
Rural health center	82 (4.2)		0.0–13.1		8.2 (8.2–8.2)		Not calculated		8.7 (1.3–16.0)	Not calculated
Reason for visit										
Fever or respiratory	548 (28.2)		22.8–33.5		12.9 (6.6–19.2)		1.0 (0.6–1.7)		9.0 (3.5–14.4)	1.8 (1.0–3.1)
COVID-19 testing	45 (2.3)		0.0–4.7		27.5 (17.7–37.3)		2.2 (1.3–3.9)		0.0 (0.0–0.0)	Not calculated
Other acute complaint	538 (27.6)		22.1–33.2		10.7 (5.6–15.7)		0.9 (0.5–1.5)		7.1 (4.6–9.7)	1.4 (0.9–2.2)
Routine health visit	368 (18.9)		9.9–27.9		12.5 (4.6–20.3)		Referent		5.0 (2.0–8.1)	Referent
Not stated	448 (23.0)		15.3–30.0		15.5 (9.8–21.2)		1.2 (0.7–2.3)		7.5 (2.6–12.4)	1.5 (0.8–2.9)
Medical history‡										
Any underlying condition	664 (34.1)		29.6–38.7		11.4 (5.6–17.1)		0.8 (0.6–1.1)		7.3 (4.3–10.3)	1.0 (0.7–1.4)
HIV	247 (12.8)		9.8–15.8		13.3 (4.3–22.4)		1.0 (0.6–1.5)		4.6 (1.3–8.0)	0.6 (0.3–1.2)
Currently pregnant¶	118 (12.5)		7.9–17.0		13.4 (3.3–23.5)		1.1 (0.7–1.9)		4.7 (0.0–11.9)	0.7 (0.2–2.6)
Hypertension	196 (10.1)		7.6–12.5		14.1 (6.9–21.3)		1.1 (0.8–1.4)		7.7 (1.2–14.2)	1.1 (0.5–2.3)
Malaria	143 (7.4)		3.2–11.6		4.8 (0.8–8.7)		0.3 (0.2–0.7)		11.1 (3.5–18.7)	1.5 (0.8–2.7)
Asthma	51 (2.6)		1.9–3.3		13.9 (0.0–28.0)		1.0 (0.4–2.8)		3.8 (0.0–11.2)	0.5 (0.1–3.4)
Diabetes	50 (2.6)		1.4–3.8		12.8 (1.8–23.9)		1.0 (0.5–1.9)		10.9 (0.0–24.8)	1.5 (0.5–4.7)
Immunocompromised	48 (2.5)		0.4–4.5		25.9 (0.0–54.8)		2.0 (0.9–4.3)		9.7 (0.0–24.5)	1.3 (0.3–5.5)
Tuberculosis	36 (1.9)		1.1–2.6		14.8 (2.4–27.2)		1.1 (0.5–2.4)		6.0 (0.0–18.5)	0.8 (0.1–4.8)
Cardiac disease	29 (1.5)		0.4–2.6		8.0 (0.2–15.8)		0.6 (0.2–1.6)		20.3 (0.0–41.5)	2.9 (1.2–6.7)
Chronic kidney disease	10 (0.5)		0.1–0.9		0.0		Not calculated		0.0	Not calculated
Emphysema/COPD	5 (0.3)		0.0–0.7		0.0		Not calculated		0.0	Not calculated
Cancer	5 (0.3)		0.0–0.7		0.0		Not calculated		0.0	Not calculated
Cirrhosis or fatty liver	1 (0.1)		0.0–0.2		0.0		Not calculated		0.0	Not calculated
Other chronic condition	83 (4.5)		2.3–6.6		6.8 (0.6–13.0)		0.5 (0.2–1.1)		11.3 (2.1–20.5)	1.5 (0.7–3.1)
Don't know	357 (18.3)		10.8–25.9		13.4 (6.8–20.1)		1.0 (0.6–1.6)		6.7 (3.0–10.3)	0.9 (0.5–1.5)
Contact with a confirmed case of COVID-19										
No	1,704 (87.7)		83.0–92.5		12.4 (7.7–17.2)		Referent		7.1 (4.3–10.0)	Referent
Yes	42 (2.2)		0.4–3.9		20.0 (2.6–37.5)		1.6 (0.8–3.4)		16.4 (6.5–26.3)	2.3 (1.4–3.8)
Don't know	196 (10.1)		5.8–14.4		18.9 (10.4–27.4)		1.5 (1.2–1.9)		6.9 (2.4–11.3)	1.0 (0.6–1.7)
Travel										
International	15 (0.8)		0.3–1.2		35.7 (8.4–63.0)		2.7 (1.5–4.8)		0.0	Not calculated
Domestic	313 (16.3)		11.3–21.4		12.4 (7.4–17.3)		0.9 (0.6–1.3)		4.5 (0.6–8.4)	0.6 (0.2–1.5)
None	1,547 (80.7)		75.9–85.6		13.4 (7.6–19.1)		Referent		7.9 (4.4–11.5)	Referent
Don't know	41 (2.1)		0.0–4.7		7.7 (0.0–19.3)		0.6 (0.1–2.4)		0.0	Not calculated
In-person work attendance, past month										
No	1,448 (75.3)		68.4–82.1		12.6 (7.9–17.2)		Referent		7.3 (4.1–10.6)	Referent
Yes	426 (22.1)		14.6–29.7		16.0 (6.0–26.0)		1.3 (0.8–2.0)		8.5 (3.4–13.7)	1.2 (0.7–2.1)
Don't know	50 (2.6)		0.0–5.5		18.2 (1.4–35.0)		1.4 (0.6–3.4)		0.0	Not calculated
Visits to the market, past month										
0–1	400 (22.2)		17.0–27.4		8.8 (3.6–14.0)		Referent		10.6 (5.4–15.7)	Referent
≥2	1401 (77.8)		72.6–83.0		14.4 (8.5–20.3)		1.6 (1.1–2.5)		6.5 (3.7–9.3)	0.6 (0.5–0.8)
Usual transportation										
Car	178 (9.3)		4.6–13.9		18.6 (12.4–24.7)		1.5 (1.0–2.2)		14.8 (5.8–23.7)	2.3 (1.1–4.7)
Taxi	215 (11.2)		4.8–17.5		15.5 (1.4–29.6)		1.2 (0.6–2.5)		4.9 (1.1–8.7)	0.8 (0.3–1.8)
Bike	63 (3.3)		1.3–5.3		4.7 (0.0–11.6)		0.4 (0.1–1.6)		6.8 (0.0–20.7)	1.1 (0.2–5.5)
Minibus	511 (26.6)		15.1–38.1		12.3 (7.4–17.2)		1.0 (0.6–1.5)		7.7 (5.0–10.5)	1.2 (0.7–2.0)
Walking	938 (48.8)		39.3–58.3		12.8 (6.7–18.9)		Referent		6.4 (2.6–10.2)	Referent
Don't know	17 (0.9)		0.0–1.8		25.0 (0.0–59.2)		2.0 (0.6–6.8)		0.0	Not calculated

*COPD, chronic obstructive pulmonary disease; COVID-19, coronavirus disease; NA, not applicable; PR, prevalence ratio; rPCR, real-time PCR †ELISA prevalence estimates and 95% CIs were adjusted for imperfect assay test characteristics (sensitivity 64.2%, specificity 100%). ‡For medical history, “no” responses were omitted from the table. ¶Analysis of pregnancy variable was restricted to women 15–49 y of age, considered the child-bearing age range in the Zambia Demographic Health Surveys and other surveys in Zambia.

SARS-CoV-2 rPCR-measured prevalence was 13.4% (95% CI 8.3%–18.5%; Table 1); SARS-CoV-2 ELISA-measured prevalence was 8.2% (95% CI 5.1%–11.4%). Compared with community members, outpatients overall had higher rPCR-measured prevalence (PR 1.8, 95% CI 1.1–2.9; Table 2) as did those seeking COVID-19 testing (PR 3.6, 95% CI 2.2–5.9) or those without a stated reason for the visit (PR 2.0, 95% CI 1.2–3.3). Although only 2.2% of participants reported contact with confirmed COVID-19 case-patients, rPCR-measured prevalence was higher among outpatients specifically seeking COVID-19 testing compared with outpatients attending facilities for another reason (PR 2.2, 95% CI 1.4–3.3). In addition, outpatients had higher ELISA-measured prevalence than community members (PR 2.5, 95% CI 1.4–4.5) (Appendix). Among outpatients with SARS-CoV-2 infection, 45.7% did not report experiencing any symptoms associated with SARS-CoV-2.

**Table 2:** Severe acute respiratory syndrome coronavirus 2 prevalence measured by PCR, prevalence ratios, and absolute prevalence differences between community members and outpatient participants, stratified by reason for attending the health facility, Zambia, July 2020

Population	Prevalence, % (95% CI)	Prevalence ratio (95% CI)	Absolute difference, % (95% CI)
Community members,† n = 2,990	7.6 (4.7–10.6)	Referent	Referent
Outpatients, n = 1,490			
Overall	13.4 (8.3–18.5)	1.8 (1.1–2.9)	5.7 (0.3–11.2)
Fever or respiratory complaint	12.9 (6.6–19.2)	1.7 (0.9–3.0)	5.3 (–1.2 to 11.7)
COVID-19 testing	27.5 (17.7–37.3)	3.6 (2.2–5.9)	19.9 (10.5–29.3)
Other acute medical complaints	10.7 (5.6–15.7)	1.4 (0.8–2.5)	3.0 (–2.4 to 8.4)
Routine health visit	12.5 (4.6–20.3)	1.6 (0.8–3.2)	4.8 (–2.9 to 12.5)
Not specified	15.5 (9.8–21.2)	2.0 (1.2–3.3)	7.9 (2.0–13.8)

*COVID-19, coronavirus disease. †Estimates derived from a cluster-sampled household prevalence survey conducted among community members in the same 6 districts (Kabwe, Livingstone, Lusaka, Nakonde, Ndola, and Solwezi) as in the outpatient prevalence study.

## Conclusions

Outpatients had higher SARS-CoV-2 prevalence than did community members in Zambia. Given the high SARS-CoV-2 prevalence and proportion of asymptomatic infections among outpatients, without instituting protective measures facilities might become transmission foci. Ameliorating risk requires instituting robust prevention and control strategies including universal masking in facilities (*9*,*10*). Furthermore, persons seeking testing at facilities should be quickly identified and isolated, because they might be at particularly high risk for having the virus.

One limitation of our study is that underlying condition and exposure history are subject to self-report and recall biases. The districts and facilities were not randomly selected and, despite our intentions to remain unbiased, may not have been representative of the population. The small sample size may have affected our ability to detect differences in factors associated with SARS-CoV-2 prevalence. The higher ELISA-measured prevalence among outpatients than community members could signal noncomparability between these 2 populations or that being an outpatient is a possible marker for other behaviors that increase SARS-CoV-2 infection risk. We assumed exact sensitivity and specificity values for the serology assay, but emerging evidence on serologic cross-reactivity (*11*–*13*) and antibody decay (*14*) might affect these values. However, given the timing of our study early in the outbreak, antibody decay was unlikely to substantially affect sensitivity (J. Perez-Saez, unpub. data, https://doi.org/10.1101/2021.03.16.21253710).

Countries with limited testing capacity need efficient strategies to identify persons with SARS-CoV-2 infections to interrupt transmission. In Zambia, when measured by rPCR, outpatients had 80% higher SARS-CoV-2 prevalence than persons in the surrounding community. Testing all outpatients regardless of their reasons for visiting the facility during periods of community transmission might help identify otherwise undetected SARS-CoV-2 infections. Compared with community-based SARS-CoV-2 testing, outpatient testing, which is often more convenient, might identify cases more effectively. Therefore, testing at facilities during periods of high community transmission might be an effective strategy to identify persons with SARS-CoV-2 infection, especially when testing capacity is limited.

Appendix Additional methods and data from a study of SARS-CoV-2 prevalence among outpatients during community transmission in Zambia, July 2020.
