# Supplementary material for: SARS-CoV-2 Prevalence among Outpatients during Community Transmission, Zambia, July 2020
Source: Emerg Infect Dis. 2021 Aug;27(8):2166–8. doi: 10.3201/eid2708.210502 (PMC8314838; doi:10.3201/eid2708.210502)
Supplement: Appendix — Additional methods and data from a study of SARS-CoV-2 prevalence among outpatients during community transmission in Zambia, July 2020. [file 21-0502-Techapp-s1.pdf]

Article DOI: https://doi.org/10.3201/eid2708.210502

SARS-CoV-2 Prevalence among Outpatients during Community Transmission, Zambia, July 2020

Appendix

Supplemental Methods

We calculated prevalence estimates using SURVEY procedures in SAS version 9.4 (SAS Institute, https://www.sas.com). The raw seroprevalence estimate was 5.3% (95% CI: 3.3%–7.3%). We adjusted seroprevalence and 95% CI estimates from SAS for imperfect assay characteristics using an R (R-project, https://www.r-project.org) function for the Rogen-Gladen estimator equation (*1*,*2*):

$$Adjusted\ prevalence = \frac{Crude\ prevalence\ +\ (Specificity - 1)}{Specificity +\ (Sensitivity - 1)}$$

```
# Function to adjust prevalence estimate according to Rogen-Gladen equation
# Specify the dataset, sensitivity, and specificity
# ‘crude’ is a data frame with 4 columns: variable name, crude prevalence,
# lower confidence limit, upper confidence limit
# ‘sensitivity’ and ‘specificity’ are objects with values for the assay’s
# sensitivity and specificity
Adjust<-function(crude,sensitivity,specificity){
  adjusted<<-(((crude[1:nrow(crude),2:4]/100)+specificity-
1)/(sensitivity+specificity-1))*100
  adjusted<<-cbind(crude[,1],adjusted)
  print(adjusted)
}
```

The Euroimmun SARS-CoV-2 IgG assay is a semiquantitative ELISA for detecting SARS-CoV-2 spike protein subunit 1 IgG in human serum or plasma. For the Euroimmun assay, a U.S. Food and Drug Administration validation study reported a sensitivity of 90% and a

specificity of 100% (*3*). However, the real world performance of this assay might be lower (*4*,*5*). An in-country validation study of Euroimmun was not performed in Zambia. As an alternative, we used sensitivity (64.2%) and specificity (100%) estimates for Euroimmun spike IgG assay from a unpublished study from Nigeria (Laura Steinhardt, personal communication) as the most applicable data for adjusting the seroprevalence estimate in our study. The unpublished study from Nigeria was conducted at the Center for Human Virology and Genomics at the Nigeria Institute of Medical Research (NIMR) in Lagos. The negative panel consisted of 50 HIV-positive samples and 49 hepatitis B surface antigen–positive samples collected and archived before October 2019. The positive panel consisted of 96 convalescent plasma specimens collected from outpatients ≥18 years testing PCR positive for SARS-CoV-2 at NIMR. Although data are emerging on factors that may impact serologic assay sensitivity and specificity by geographic locations or by the length of time following infection (*6–10;* J. Perez-Saez, unpub. data, https://doi.org/10.1101/2021.03.16.21253710), for the purpose of this analysis we assumed the exact values and applied the transformation to the upper and lower CI bounds.

References

1. Sempos CT, Tian L. Adjusting coronavirus prevalence estimates for laboratory test kit error. Am J Epidemiol. 2021;190:109–15. PubMed https://doi.org/10.1093/aje/kwaa174

2. Stevenson M, Sergeant E, Nunes T, Heuer C, Marshall J, Sanchez J, et al. EpiR package. 2021. https://cran.r-project.org/web/packages/epiR/epiR.pdf

3. U.S. Food and Drug Administration. Serology test evaluation report for “SARS-COV-2 ELISA (IgG)” from Euroimmun. 2020. [cited 27 May 2021] https://www.accessdata.fda.gov/cdrh_docs/presentations/maf/maf3246-a001.pdf

4. Nkuba Ndaye A, Hoxha A, Madinga J, Mariën J, Peeters M, Leendertz FH, et al. Challenges in interpreting SARS-CoV-2 serological results in African countries. Lancet Glob Health. 2021;9:e588–9. PubMed https://doi.org/10.1016/S2214-109X(21)00060-7

5. Yadouleton A, Sander A-L, Moreira-Soto A, Tchibozo C, Hounkanrin G, Badou Y, et al. Limited specificity of serologic tests for SARS-CoV-2 antibody detection, Benin. Emerg Infect Dis. 2021;27:233–7. PubMed https://doi.org/10.3201/eid2701.203281

6. Tso FY, Lidenge SJ, Peña PB, Clegg AA, Ngowi JR, Mwaiselage J, et al. High prevalence of pre-existing serological cross-reactivity against SARS-CoV-2 in sub-Sahara Africa. Int J Infect Dis. 2020;102:577–83. PubMed https://doi.org/10.1016/j.ijid.2020.10.104

7. Choe PG, Kim K-H, Kang CK, Suh HJ, Kang E, Lee SY, et al. Antibody responses 8 months after asymptomatic or mild SARS-CoV-2 infection. Emerg Infect Dis. 2021;27:928–31. PubMed https://doi.org/10.3201/eid2703.204543

8. Choe PG, Kang CK, Suh HJ, Jung J, Song KH, Bang JH, et al. Waning antibody responses in asymptomatic and symptomatic SARS-CoV-2 infection. Emerg Infect Dis. 2021;27:327–9. PubMed https://doi.org/10.3201/eid2701.203515

9. Steinhardt LC, Ige F, Iriemenam NC, Greby SM, Hamada Y, Uwandu M, et al. Cross-reactivity of two SARS-CoV-2 serological assays in a malaria-endemic setting. J Clin Microbiol. 2021; JCM.00514–21. [online ahead of print]

10. Wiens KE, Mawien PN, Rumunu J, Slater D, Jones FK, Moheed S, et al. Seroprevalence of Severe Acute Respiratory Syndrome Coronavirus 2 IgG in Juba, South Sudan, 2020. Emerg Infect Dis. 2021;27:1598–606. 10.3201/eid2706.210568 PubMed https://doi.org/10.3201/eid2706.210568

11. Mulenga LB, Hines JZ, Fwoloshi S, Chirwa L, Siwingwa M, Yingst S, et al. Prevalence of SARS-CoV-2 in six districts in Zambia in July, 2020: a cross-sectional cluster sample survey. Lancet Glob Health. 2021;9:e773–81. PubMed https://doi.org/10.1016/S2214-109X(21)00053-X

Appendix Table 1. Distribution and inclusion of health care facilities of sample by district*

	Districts						
Category	Kabwe	Livingstone	Lusaka	Nakonde	Ndola	Solwezi	Total
Total health facilities by district	395	266	652	156	330	257	2,056
No. health facilities in study by district	3	3	6	2	3	3	20
% Health facilities in study from each district	19.2%	12.9%	31.7%	7.6%	16.1%	12.5%	100%

*3 health facilities were COVID-19 treatment centers in Kabwe, Livingstone, and Lusaka

Appendix Table 2. Differences in SARS-CoV-2 prevalence measured by ELISA between community members and outpatient participants, stratified by reason for attending the health facility.

Population		Prevalence, % (95% CI)	Prevalence ratio (95% CI)
Community members† (n = 2,704)		3.2 (1.7–4.8)‡	Referent
Outpatients (n = 1,490)	Overall	8.2 (5.1–11.4)	2.5 (1.4–4.5)
	Fever or respiratory complaint	9.0 (3.5–14.4)	3.4 (1.8–6.6)
	COVID-19 testing	0.0	Not calculated
	Other acute medical complaints	7.1 (4.6–9.7)	2.3 (1.3–4.0)
	Routine health visit	5.0 (2.0–8.1)	1.7 (0.8–3.6)
	Not specified	7.5 (2.6–12.4)	2.6 (1.2–5.4)

*COVID-19, coronavirus disease
†Estimates derived from a cluster-sampled household prevalence survey conducted among community members in the same 6 districts (Kabwe, Livingstone, Lusaka, Nakonde, Ndola, and Solwezi) as in the outpatient prevalence study (*11*).
‡Estimate differs from the published estimate of 2.1% (95% CI 1.1%–3.1%) because it has been adjusted for the imperfect test characteristics of the Euroimmun assay using the Rogen-Gladen estimator equation.

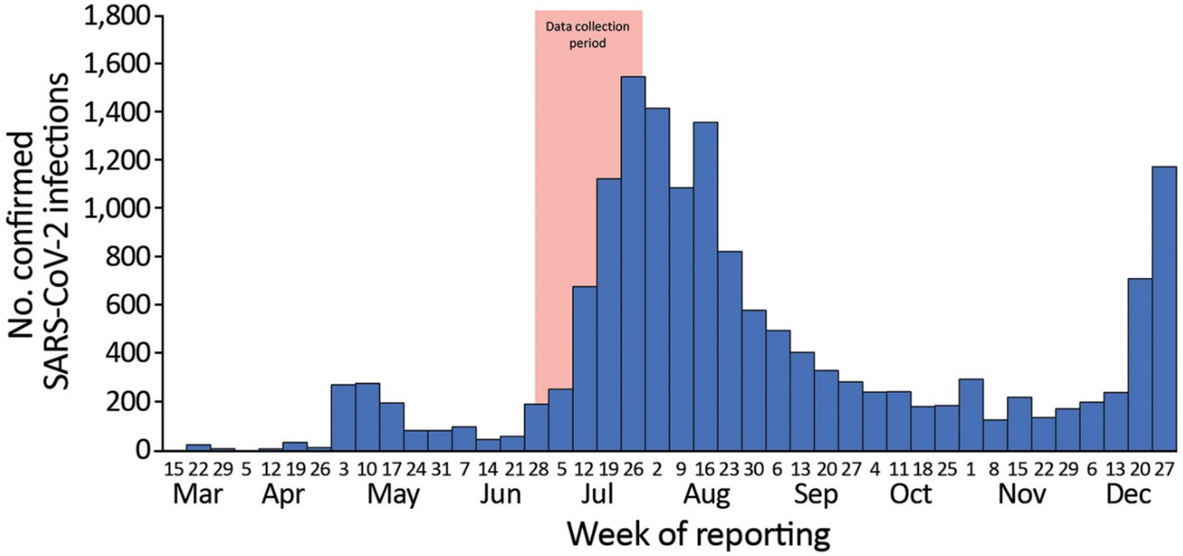

Appendix Figure 1. Confirmed SARS-CoV-2 infections by week of laboratory confirmation in six districts (Kabwe, Livingstone, Lusaka, Nakonde, Ndola, and Solwezi) in Zambia, March–December 2020. Outpatients survey data was collected July 2–31, 2020, household survey data was collected July 4–27, 2020.

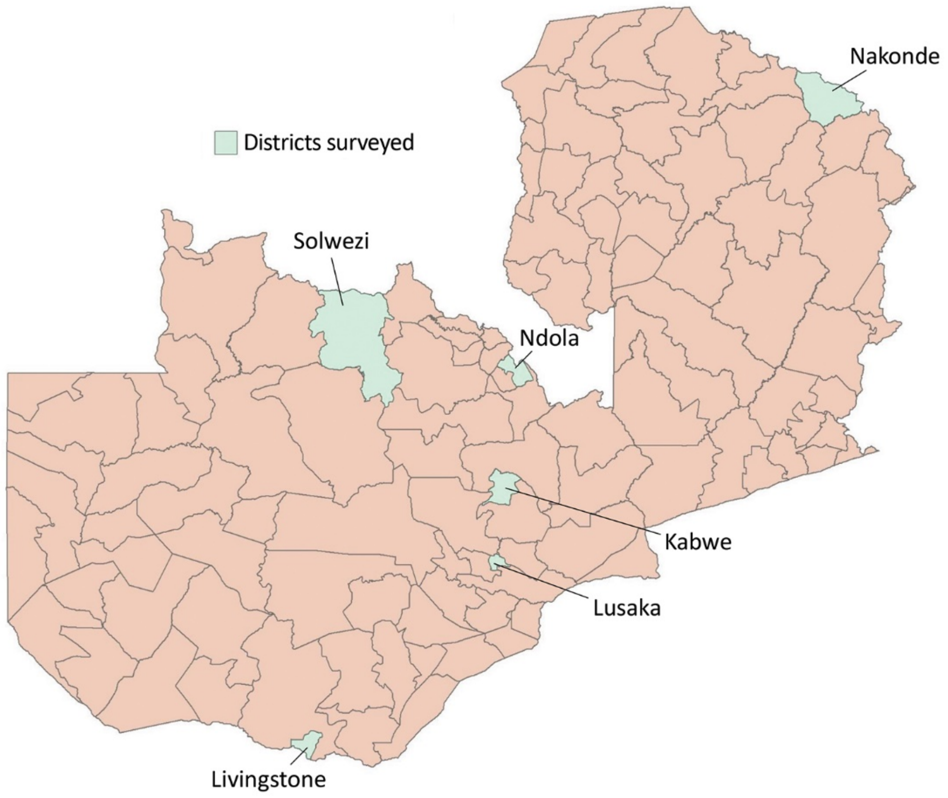

Appendix Figure 2. District locations for SARS-CoV-2 prevalence study among outpatients in six districts (Kabwe, Livingstone, Lusaka, Nakonde, Ndola, and Solwezi) in Zambia, July 2020.

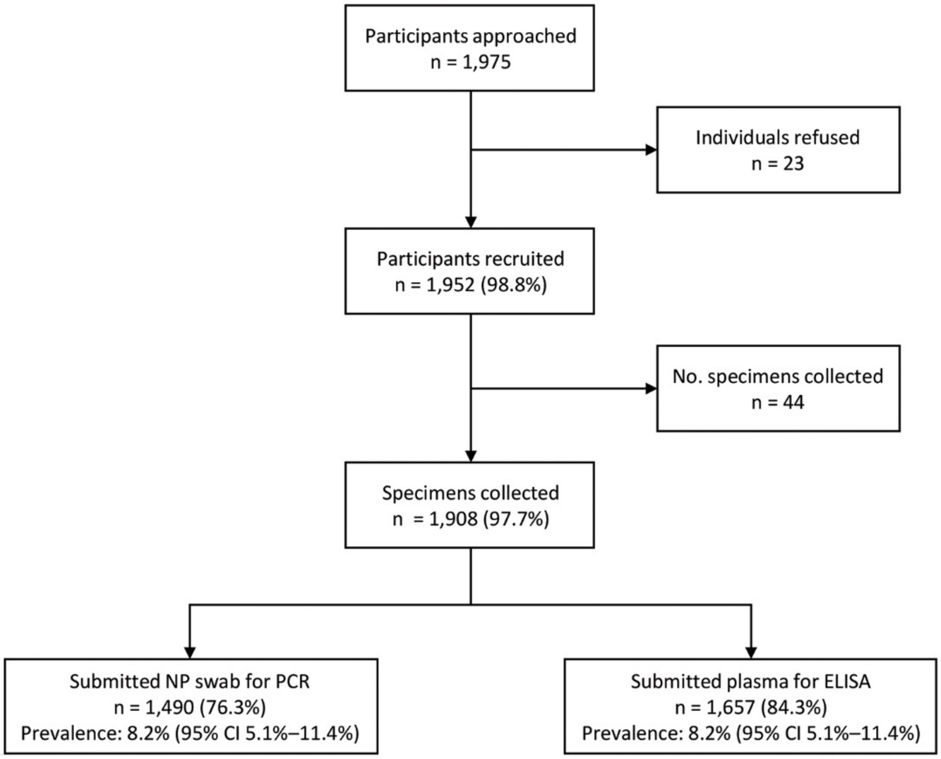

Appendix Figure 3. SARS-CoV-2 prevalence study flow diagram with sample sizes, response rates, and prevalence estimates in six districts (Kabwe, Livingstone, Lusaka, Nakonde, Ndola, and Solwezi) in Zambia, July 2020.
